# WebAUGUSTUS—a web service for training AUGUSTUS and predicting genes in eukaryotes

**DOI:** 10.1093/nar/gkt418

**Published:** 2013-05-21

**Authors:** Katharina J. Hoff, Mario Stanke

**Affiliations:** Institute for Mathematics and Computer Science, Ernst Moritz Arndt University, 17487 Greifswald, Germany

## Abstract

The prediction of protein coding genes is an important step in the annotation of newly sequenced and assembled genomes. AUGUSTUS is one of the most accurate tools for eukaryotic gene prediction. Here, we present WebAUGUSTUS, a web interface for training AUGUSTUS and predicting genes with AUGUSTUS. Depending on the needs of the user, WebAUGUSTUS generates training gene structures automatically. Besides a genome file, either a file with expressed sequence tags or a file with protein sequences is required for this step. Alternatively, it is possible to submit an externally generated training gene structure file and a genome file. The web service optimizes AUGUSTUS parameters and predicts genes with those parameters. WebAUGUSTUS is available at http://bioinf.uni-greifswald.de/webaugustus.

## INTRODUCTION

The structural annotation of protein coding genes serves as a basis for many further steps in the analysis of sequenced and assembled eukaryotic genomes. Many of the currently available gene prediction tools rely on stochastic models incorporating usually several thousands of parameters. These parameters need to be adapted to species-specific traits to achieve most accurate gene prediction results. With the exception of self-training methods [e.g. the command line tool GeneMark-ES (1)], parameters are usually adapted using a set of already annotated genes in the target genome; here, we refer to those gene structures as *training genes*.

Training genes can for instance be built from alignments of expressed sequence tags (ESTs) or protein sequences against the target genome. For example, the initial training genes for annotating the genome of *Amphimedon queenslandica* were generated from EST alignments (2), and protein sequences were used for generating training genes for *Coprinus cinerea* (3).

Scripts or tutorials for training gene finders are often available for non-commercial gene finders. A skilled bioinformatician should currently for example be able to train and execute SNAP (4), AUGUSTUS (5,6), mGene (7) and GeneID (8). However, installing required software and training a gene prediction tool can be difficult for inexperienced users, as basic programming skills are often required (9).

This problem was already recognized by Schweikert *et al.* (10), who developed mGene.web. Their web service was supposed to enable software users without programming skills to train and execute the gene prediction tool mGene. However, at the time of submission of this manuscript, mGene.web was temporarily not fully functional because it was being migrated to a new sever.

Independent accuracy assessments have shown that AUGUSTUS belongs to the most accurate gene finding tools (11,12). Training AUGUSTUS for a novel species has up to now been challenging for many users. Therefore, we here describe WebAUGUSTUS, a web service for automated training gene structure generation, training AUGUSTUS and predicting genes with AUGUSTUS.

## WEB SERVICE

WebAUGUSTUS provides two web interfaces:

1. *AUGUSTUS Training* generates training gene structures, trains AUGUSTUS and predicts genes with AUGUSTUS in a fully automated way.
2. *AUGUSTUS Prediction* predicts genes with AUGUSTUS in genomic sequences using already trained parameters.

### AUGUSTUS Training

To date, AUGUSTUS has been trained by experts for >50 species. The parameter files are publicly available and can be applied across program versions of AUGUSTUS for predicting genes in genomic sequences of those 50 species and for species that are not distantly related to all of those 50 species. The *AUGUSTUS Training* web server application can be used to optimize AUGUSTUS parameters for novel species.

#### Input

The *AUGUSTUS Training* web interface offers three different data set options for training AUGUSTUS (further referred to as optA, optB and optC), which require different input file combinations:

optA Fully automated training gene structure generation on the basis of a genome and a cDNA file requires both files in fasta format.
optB Fully automated training gene structure generation on the basis of a genome and protein sequence file requires both files in fasta format.
optC Already existing training gene structures can be submitted in gene transfer format (gtf) or genbank format in addition to a genome file in fasta format.

Genome files should contain high-quality genomic sequences (long scaffolds or contigs that can encode for complete genes are required). cDNA files may contain ESTs or assembled full-length cDNA sequences. Protein files should contain full-length protein sequences. We recommend that cDNA files and protein files originate from the same species as the target genome file. In some cases, data from close relatives (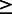
95% protein sequence identity) might also lead to good results, but more frequently, it is not possible to generate a sufficient amount of training genes from using another’s species proteins. Externally prepared training gene structures should contain complete gene structures, only (i.e. no parts of the coding sequence should be missing).

#### Software

The *AUGUSTUS Training* web service provides an interface to a Perl pipeline called AutoAug.pl (available at http://bioinf.uni-greifswald.de/augustus/binaries/scripts).

If invoked with data from optA, autoAug.pl assembles cDNA sequences into gene structures using PASA (13). AUGUSTUS parameters are optimized using those gene structures. After successful training, *ab initio* gene prediction in the genome file is performed. Subsequently, the cDNA sequences are used to create hints for AUGUSTUS using BLAT (14), and genes are predicted using the extrinsic cDNA information as described previously (6). If possible, training examples for untranslated regions (UTRs) are assembled from cDNA information and predicted protein coding regions, and UTR-parameters for AUGUSTUS are trained. After this second training step, genes are predicted with the beforehand created hints and UTRs.

In case of data from optB, Scipio (15) is used to generate training gene structures from alignments of protein sequences to the genome. AUGUSTUS parameters are optimized using those gene structures. After successful training, *ab initio* gene prediction in the genome file is performed.

If data from optC is submitted, AUGUSTUS parameters are trained, and meta parameters, such as splice site window sizes, are optimized using the provided training gene structures. Afterwards, genes are predicted *ab initio* in the genome sequences.

It is also possible to submit a genome file, a cDNA file and a protein file. In that case, the same steps as in case optB are performed, but in addition, hints are created from the cDNA sequences as described in optA, and genes are predicted with this extrinsic evidence.

Reasonable training of AUGUSTUS parameters will require at least several hundreds of training gene structures. WebAUGUSTUS will not start training with ≤100 training gene structures.

#### Output

One major goal of the *Training* web interface is to return parameters that are optimized for predicting genes in a genomic sequence of a species of interest with AUGUSTUS. These can be used without retraining to predict genes when new assemblies or new transcript sequences are available. In addition, gene predictions according to the different workflows described for optA, optB and optC will be returned if possible.

All jobs will return a log-file AutoAug.log and an error-file AutoAug.err. It is generally recommended that users inspect these files before they continue to work with other results files.

If AUGUSTUS training was possible, an archive parameters.tar.gz with AUGUSTUS parameters is returned. After download and extraction, this archive can be used within a local AUGUSTUS installation. In addition, parameter sets that were trained via the *AUGUSTUS Training* web interface are immediately available to the *AUGUSTUS Prediction* web service for future predictions. The web service also returns compressed training gene structures in genbank format that were used for optimizing the parameter set (training.gb.gz).

In case of successful gene prediction, compressed gene prediction archives are returned. Possible gene prediction archives are listed in Table 1. *Ab initio* gene prediction will always be performed after successful training. Predictions with hints will only be performed if a cDNA file was provided, and if it was possible to generate hints from aligning the contained sequences against the genome. Predictions with UTRs will only be provided if it was previously possible to train UTR parameters for AUGUSTUS.

**Table 1.** Compressed gene prediction archives of *AUGUSTUS Training*

Archive name	Description
ab_initio.tar.gz 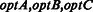	*Ab initio* predictions
pred_hints.tar.gz*^optA^*	Predictions with hints from cDNAs
pred_hints_utr.tar.gz*^optA^*	Predictions with UTRs and hints from cDNAs

All successful runs of *AUGUSTUS Training* will return an *ab initio* archive. The other two archives are only returned if a cDNA file for hint generation was provided. Predictions with UTR are only possible after successful UTR parameter training.

All gene prediction archives contain at least one file in general feature format (gff). If no genes were predicted, this will be the only file. If it was possible to predict genes, gene structures are also contained in gtf- and gbrowse-format. Furthermore, predicted amino acid sequences, coding sequences and exons of coding sequences in fasta format are then contained in a prediction archive. If UTR parameter optimization was possible, the predicted mRNA sequences are additionally contained in fasta format. Table 2 summarizes files that can be contained in gene prediction archives.

**Table 2.** Compressed gene prediction output archives generated by WebAUGUSTUS may contain files with the following file endings

File name ending	Description
*.gff	Predictions in gff format
*.gtf^a^	Predictions in gtf format
*.aa^a^	Predicted amino acid sequences in fasta format
*.codingseq^a^	Predicted coding sequences in fasta format
*.cdsexons^a^	Predicted exon sequences in fasta format
*.mrna^a,^^b^	Predicted mRNA sequences in fasta format
*.gbrowse^a^	Gene predictions formatted as a track for GBrowse

^a^Files are only produced if at least one gene was predicted.

^b^File is only produced if it was possible to train UTR parameters for AUGUSTUS, and in case of AUGUSTUS Prediction, only if UTR prediction was explicitly enabled.

### AUGUSTUS Prediction

The new *AUGUSTUS Prediction* web service is directly connected to a database that stores species-specific parameters that were trained by using the *Training* web service, i.e. if a user has trained AUGUSTUS parameters via WebAUGUSTUS, those parameters are instantly available for predicting genes in more genomic sequences by using the *AUGUSTUS Prediction* webinterface.

#### Input

Gene predictions are performed in a provided genome file in fasta format. Additionally, a parameter set must be specified (either via training job ID or by selection from a drop-down menu with expert-trained parameters, or by uploading an externally trained parameter archive). Parameter sets that were trained using WebAUGUSTUS are identified via an ID (trainxxxxxxxx) that is only available to the user who performed the training (the chances of guessing the parameter ID of a training job are roughly 1:10^14^).

Optionally, users may upload a file with cDNA sequences that will be used to automatically generate hints or an externally created hints file in AUGUSTUS-specific gff-format.

Checkboxes offer the functionality of enabling UTR prediction (only possible if UTR parameters for the species in question exist), reporting genes on certain strands, enabling alternative transcripts and allowed predicting gene structures (e.g. only complete genes or complete and partial genes).

#### Software

If a cDNA file was provided, WebAUGUSTUS will use BLAT to align the cDNA sequences to the genome. The alignments are converted to hints. In any case, AUGUSTUS will be executed with arguments that match the user-specified requirements. In contrast to the *Training* web service, which automatically tries to run many subsequent prediction steps, the *AUGUSTUS Prediction* web service will run only exactly one gene prediction job at a time.

#### Output

After WebAUGUSTUS has finished a gene prediction job, prediction results will be available for download in a compressed archive that contains at least a gff-file, but may optionally also contain the other files listed in Table 2.

### Implementation

AUGUSTUS and parts of the training routine are implemented in C++, the wrapping pipeline is implemented in Perl. The web service is implemented in Grails. Submitted jobs are scheduled via a Sun Grid Engine. Currently, eight jobs can be executed in parallel.

According to its license, BLAT is freely available for academic, non-profit and personal use. Commercial users are, therefore, not allowed to use WebAUGUSTUS for processes that involve the usage of BLAT. That means, commercial users are only allowed to run *AUGUSTUS Training* with externally generated training gene structures, and the submission of cDNA files is not allowed for commercial users in *AUGUSTUS Training* and *Prediction*.

## MATERIALS AND METHODS

Prediction accuracy with parameters trained by WebAUGUSTUS and by human experts was measured using three different data sets. For optA, the genome of the insect *Drosophila melanogaster* (assembly BDGP R5/dm3) and 818 005 ESTs from the same species that were obtained from the National Center for Biotechnology Information (NCBI) were used. OptB was evaluated using the genome of the plant *Arabidopsis thaliana* (assembly TAIR 10) and 35 375 protein sequences of the same species that were obtained from NCBI. OptC was evaluated using the genome of the worm *Caenorhabditis elegans* and 18 555 training gene structures retrieved from Wormbase (16).

To avoid an overly optimistic performance estimate for the new genes, the chromosomes of all genomes [for fly and plant downloaded from the UCSC Genome Browser database (17)] were split into two parts in such a way that ∼50% of the genes were located on the first half, and the remaining genes were located on the second half. The second part of all chromosomes was used as a genomic input sequence for training AUGUSTUS, whereas the first part served for accuracy assessment opf gene predictions.

For *D.melanogaster*, protein coding genes from FlyBase (18), for *A. thaliana*, protein coding genes from TAIR 10 (19) and for *C. elegans*, protein coding genes from Wormbase were used as a reference annotation for measuring accuracy.

The exact source of all data sets and the files used for the actual experiments are described in detail in Supplementary Materials, section Supplementary Methods: Data Sets.

Commonly used measures of accuracy (measured in percent) in gene prediction are

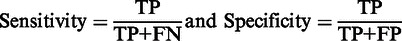

where TP stand for true positives, i.e. the number of predicted features that agree with the gold-standard reference, FN stands for false negatives, i.e. the number of features that were overseen by the predictor and FP stands for false positives, i.e. the number of features that were predicted but not in agreement with the reference annotation.

Sensitivity and specificity were measured for the features gene (i.e. only a gene structure that was predicted correctly including the exact positions of all CDS exons was counted as TP), exon (i.e. only exons that were predicted correctly were counted as TP) and nucleotide (i.e. every correctly predicted nucleotide was counted as TP).

## RESULTS

*Ab initio* gene prediction accuracy results from training and gene prediction via WebAUGUSTUS are shown in Table 3. Additionally, we show gene prediction accuracy obtained with parameter sets that were trained by experts. Here, performance depends on the amount and quality of input data. The here reported differences between expert and automated training are small. For optA, it should be noted that the expert-trained parameters contained customized modifications for the particular case of *D. melanogaster* that are not possible via WebAUGUSTUS (e.g. the length of donor and acceptor splice sites was altered). In case of optC, accuracy obtained by using WebAUGUSTUS seems to be slightly higher than accuracy obtained with the expert-trained parameters. This may be explained by the fact that the web parameters were trained and tested on genes from the current Wormbase release, whereas the expert parameters were trained using an earlier annotation. In general, higher accuracy values can be expected when using the same parameter sets in combination with extrinsic evidence.

**Table 3.** *Ab initio* gene prediction accuracy results of WebAUGUSTUS with web-trained and expert-trained parameters

Scenario	Trainer	Gene level				Exon level				Nucleotide level			
		Sens.	Spec.	#Anno	#Pred	Sens.	Spec.	#Anno	#Pred	Sens.	Spec.	#Anno	#Pred
optA	web	46.3	37.2	5660	7099	67.6	57.8	24 846	29 082	90.3	69.8	9 387 473	12 157 611
	Expert	49.0	40.3		6897	71.5	56.8		31 241	93.2	67.0		13 053 363
optB	Web	56.8	45.7	13 535	16 843	82.0	72.5	73 625	83 234	96.4	76.1	16 504 394	20 906 994
	Expert	58.9	46.4		16 920	83.1	71.2		85 883	96.9	75.3		21 226 128
optC	Web	37.2	39.3	9992	9450	74.8	76.0	63 286	62 301	90.0	87.1	12 394 167	12 791 466
	Expert	32.4	36.8		8794	71.7	75.6		60 111	87.7	87.5		12 420 039

Accuracy was measured by comparing predicted genes to existing annotations. Parameters were optimized using the three different approaches that are available at WebAUGUSTUS: training AUGUSTUS with gene structures that were generated in a fully automated way from ESTs (optA, *D. melanogaster*) or protein (optB, *A. thaliana*) sequences, and training AUGUSTUS with externally generated gene structures (optC, *C. elegans*). For each scenario, we show accuracy results that were obtained using WebAUGUSTUS, and in a row below, the accuracy results obtained with already existing parameter sets that were generated by experts.

Spec., Specificity; Sens., Sensitivity; #Anno, number of annotated features; #Pred, number or predicted features.

The runtimes of training and prediction jobs that were executed for preparing the results are shown in Table 4 (jobs are executed sequentially on the server, i.e. only one CPU is allocated to each job). Although the training jobs of optB and optC required a couple of hours, only, the training job of optA required several days. Runtime depends on the size of data sets, on the executed pipeline, on the resulting number of training gene structures and on the obtained parameter set. Given the same number of resulting training gene structures, optC will always be faster than optA and optB because the training gene structure file does not need to be generated by the web service. In turn, given the same number of resulting training gene structures, optA will always be slower than optB because optB does not attempt to perform the assembly of UTR training examples and UTR parameter training. Certain properties of the sequences, in particular the number of unknown nucleotides, and parameter sets influence the execution time for gene prediction jobs, e.g. the maximal length of UTR exons can lead to different execution times because the longer a UTR exon can potentially be, the more candidates must be scored.

**Table 4.** Computational time of WebAUGUSTUS for optA, optB and optC

Scenario	Training time (min)	Prediction time (min)
optA	10 849	5283 (630)
optB	736	91 (95)
optC	351	(71)^a^

In brackets, we show the computational time that was needed to complete predictions on the same test sequences with the expert-trained parameters.

^a^Training and prediction on the two different data sets were performed by one autoAug.pl run of the Training Web Service, i.e. prediction time is included in training time.

## DISCUSSION AND CONCLUSION

WebAUGUSTUS is currently the only functional web service for generating training gene structures and training a eukaryotic gene prediction tool. In comparison, mGene.web does not offer automated training gene generation, and it requires a lot of interaction with the user (building customized workflows and so forth). In contrast to this, WebAUGUSTUS is fairly easy to use: after filling in a web form, the entire job is executed automatically.

Although not available as a web service, the usage of GeneMark-ES for one-step training and prediction is as easy to operate as a web service. However, GeneMark-ES was designed for small and not complex genomes. We would, therefore, like to mention that the gene prediction accuracy of WebAUGUSTUS in relatively complex eukaryotic genomes (e.g. *D. melanogaster*) is higher in comparison with the accuracy of GeneMark-ES (see Supplementary Materials, section Supplementary Results: Accuracy of GeneMark-ES).

Concerning the here reported accuracy results, users should be aware of the fact that accuracy of gene prediction with parameters that were optimized using WebAUGUSTUS strongly depends on the input data quality. A low number of training gene structures, or low-quality training gene structures, may lead to poor accuracy.

In comparison with the freely available binaries and scripts for execution of AUGUSTUS on a local computer, the functionality of WebAUGUSTUS is limited. For example, Conditional Random Field (CRF) training is not accessible via WebAUGUSTUS, as this method is less robust to errors in the training gene set than Generalized Hidden Markov Model training. Also the integration of RNA-Seq data and several other frequently used sources of extrinsic evidence is currently not supported by WebAUGUSTUS unless the user first prepares the hints locally. In some cases, the CRF will yield better accuracy results, and the inclusion of as much high-quality extrinsic evidence as possible will most definitely improve gene prediction accuracy.

## SUPPLEMENTARY DATA

Supplementary Data are available at NAR Online: Supplementary Methods and Supplementary Results.

## FUNDING

Funding for open access charge: Deutsche Forschungsgemeinschaft (DFG) [HO4545/1-1, STA1009/6-1], institutional funding (Institute for Mathematics and Computer Science, Ernst Moritz Arndt University of Greifswald).

*Conflict of interest statement.* None declared.
